# The Biodistribution and Utility of ^99m^Tc-Ethylenedicysteine-Deoxyglucose (^99m^Tc-Glucosamine) in the Identification of Active Disease in Patients with Rheumatoid Arthritis—a Single Center Prospective Study

**DOI:** 10.1007/s13139-023-00823-4

**Published:** 2023-09-25

**Authors:** Osayande Evbuomwan, Barend Jansen Van Rensburg, Gerrit Engelbrecht, Cathryn H. S. Driver, Mathys Labuschagne, Joseph Sempa, Je’nine Horn-Lodewyk

**Affiliations:** 1 Nuclear Medicine Department, University of The Free State, Universitas Academic Hospital, Lower Ground Floor, Logeman Street, Bloemfontein, 9301 South Africa; 2 University of The Free State, Bloemfontein, South Africa; 3 Department of Radiochemistry, South African Nuclear Energy Corporation, Pretoria, South Africa; 4 Central University of Technology, Bloemfontein, South Africa; 5 Te Whatu Ora Health, Wellington, New Zealand

**Keywords:** ^99m^Tc-ECDG, Glucosamine, Rheumatoid arthritis, SPECT/CT

## Abstract

**Purpose:**

Our objectives were to investigate the utility of ^99m^Tc-ethylenedicysteine-deoxyglucose (ECDG) in identifying active disease in the joints of patients with rheumatoid arthritis (RA), as well as to evaluate the biodistribution of this radiopharmaceutical.

**Methods:**

A prospective study was conducted at the Department of Nuclear Medicine of the University of the Free State/Universitas Academic Hospital in Bloemfontein, South Africa. Twenty-two participants from the rheumatology department diagnosed with RA according to the ACR/EULAR classification criteria were enrolled. Participants were injected with 20–25 mCi of ^99m^Tc-ECDG. Flow, blood pool, whole body, delayed static, and SPECT/CT images were acquired. Known sites of disease were qualitatively assessed for intensity of uptake, and disease severity was graded (Grade 0–3).

**Results:**

Twenty-two participants were studied. The median (interquartile range) age was 59 (49–68) years, and the majority (*n* = 21; 95.5%) were females. There was abnormal increased uptake of ^99m^Tc-ECDG noted in majority of the sites of known disease, including unknown sites. SPECT/CT imaging localized radiotracer uptake specifically to the synovial space. Similar biodistribution of radiotracer was noted in all patients, irrespective of disease severity or fasting status.

**Conclusion:**

^99m^Tc-ECDG can efficiently assess disease activity in the joints of patients with RA. It accumulates in sites of both clinical and subclinical disease and might be a very useful tool for the rheumatologist in the management of patients with RA.

## Introduction

Rheumatoid arthritis (RA) is a chronic inflammatory disease that can lead to irreversible joint damage, deformities, disability, and premature mortality, if not treated promptly and properly [1]. Therefore, prompt diagnosis and treatment will offer a very good prognosis in these patients. Bone erosion is a central feature of this pathology in patients who do not start treatment early and is associated with severe disease and poor functional outcomes [2]. Bone erosion has also been found to occur in some patients with low disease activity or those in clinical remission [2]. In order to offer early and proper treatment, investigations with a high sensitivity and specificity are needed to identify active disease.

Several advances in the methods for scoring disease activity in RA have been made in recent years. This involves a combination of the clinical assessment of involved joints and laboratory investigations. The three validated scoring systems currently used in South Africa are the 28-Joint Disease Activity Score (DAS-28), the Simplified Disease Activity Index (SDAI), and the Clinical Disease Activity Index (CDAI) [1]. These scores allow for the classification of the patient as being in remission or presenting with low, moderate, or high disease activity. However, non- specific constitutional symptoms such as fatigue and malaise may predominate in some patients [3], and the subjective nature of these assessments might reduce their sensitivity. This shortcoming has given rise to the increased use of ultrasound (US) and magnetic resonance imaging (MRI) in the evaluation of these patients, due to their higher diagnostic accuracy [3]. In short, the latest classification criteria often used to diagnose RA, proposed by the American College of Rheumatology/European League Against Rheumatism (ACR/EULAR), incorporated the role of US and MRI in the detection of synovitis, thus enabling earlier diagnosis and correct classification of patients [4, 5]. However, these imaging modalities are not without their drawbacks. The major drawback of US is that it is operator-dependent and cumbersome, especially when almost every joint in both hands must be evaluated [4, 6]. The inconvenience of this method is further increased if additional joints beside those in the hands need evaluation.

Magnetic resonance imaging has a high diagnostic accuracy in evaluating disease activity in the joints of patients with RA. It has shown strength in identifying early inflammatory changes [7, 8]. This imaging modality provides greater resolution of joint structure and pathology but has the disadvantage of being able to screen only single joints/regions at a time. Additionally, MRI is very expensive, which increases the cost of patient imaging.

In the last decade, the use of nuclear medicine imaging to diagnose diseases and provide functional information regarding physiological processes has become increasingly valuable. With respect to RA, conventional planar scintigraphy and single photon computed tomography (SPECT), as well as positron emission tomography (PET), have been applied using a number of radiopharmaceuticals for the evaluation of disease activity [9]. The use of hybrid imaging, combining PET or SPECT imaging with computed tomography (CT), has further increased the diagnostic accuracy of these imaging techniques.

Technetium-99 m (^99m^Tc) diphosphonates, specifically ^99m^Tc-methylene diphosphonate (^99m^Tc-MDP) which accumulates in bones is most commonly used for the evaluation of RA through planar bone scintigraphy and SPECT. While this method can assist in identifying arthritic joints, the disadvantage is low specificity and limited spatial resolution as compared to MRI [9].

The most commonly used PET/CT radiopharmaceutical for the evaluation of patients with RA is fluorine-18-fluorodeoxyglucose ([^18^F]FDG) [10–13]. Some studies have shown [^18^F]FDG PET/CT to have a comparable diagnostic accuracy to US and MRI [9, 10]. However, not every nuclear medicine center has a PET/CT scanner, and along with the cost and shorter half-life of [^18^F]FDG, it makes the application of this method slightly more prohibitive.

These drawbacks of the above mentioned imaging modalities show that there is still a need for a cost-effective, highly reliable, easy to perform, and highly sensitive technique for the detection of disease activity in the joints of patients with RA.

^99m^Tc-ethylenedicysteine-deoxyglucose (^99m^Tc-ECDG), also known in essence as ^99m^Tc-glucosamine (both names to be used interchangeably), is a SPECT radiopharmaceutical for functional imaging of highly metabolic cells and has been investigated for imaging of cancer, similar to [^18^F]FDG [14]. Glucosamine is an important basic natural component of cartilage and synovial fluid [15], and radiolabeled glucosamine may therefore find useful application for joint imaging and evaluation of patients with RA. Very few studies have shown the potential utility of ^99m^Tc-ECDG imaging in patients with RA [6, 14]. The aim of this study was therefore to, in a prospective manner, investigate ^99m^Tc-ECDG, within a local context, for the evaluation of active joint disease in patients with RA, including evaluation of the general whole body biodistribution of the radiopharmaceutical.

## Materials and Methods

Ethical approval for the study was obtained from our institution’s Human Research Ethics Committee.

### Study Population and Design

This was a prospective cross sectional study which was conducted at the Department of Nuclear Medicine, University of the Free State/Universitas Academic Hospital in Bloemfontein, South Africa. Twenty two participants from the rheumatology clinic were recruited into this study. These participants were diagnosed with RA by an experienced rheumatologist (25 years experience), according to the ACR/EULAR classification criteria. They had disease involving either the wrist, small bones of the hands, and the knees. Recruitment period was between February and August 2022. Signed consent was obtained from all study participants.

### Radiopharmaceutical Preparation

All commercial reagents and solvents were purchased from Sigma-Aldrich (Millipore Sigma, USA) or Merck Millipore (USA). All samples for ^1^H-NMR spectroscopy were prepared using CDC_l3_ or D_2_O and analyzed on a Bruker 300 MHz spectrometer (Massachusetts, USA). High-performance liquid chromatography (HPLC)-MS analysis was done using an Agilent Infinity 1200 Series system coupled to Agilent 6100 Series quadrupole MS system (Agilent, USA) with radiometric GABI Star gamma detector (Raytest GmbH, Straubenhardt, Germany).

### Synthesis of ECDG

The ECDG ligand was synthesized according to the procedure described by Yang et al. [16]. Briefly, L-thiazolidine- 4-carboxylic acid (30.0 g) was dissolved in liquid ammonia (150 mL) followed by the slow addition of sodium metal (8.0 g, 1.5 eq) resulting in a deep blue-colored solution. The solution was stirred for 20 min at room temperature. The reaction was quenched with ammonium chloride (5.0 g), leading to the evaporation of ammonia solvent, and the resulting residue dissolved in water (200 mL). The pH was adjusted to 3.0, and the resultant precipitate was purified by recrystallization in ethanol to yield ethylenedicysteine 2HCl (EC) (38% yield).

EC (2.0 g) was dissolved in 2 M NaOH (30 mL) with ethanol (40 mL) and stirred vigorously for 20 min. Benzyl chloride (1.48 g, 2.0 eq) in dioxane (20 mL) was added dropwise to the solution and further stirred for 30 min, after which the organic solvents were removed. The pH of the resulting aqueous mixture was acidified to pH 3.0 resulting in the precipitation of the hydrochloride salt of *S,S′*-dibenzyl ethylene dicysteine (Bn-EC) (85% yield), which was used without further purification.

Benzyl chloroformate (4.90 g, 2.5 eq) in dioxane (150 mL) was added to a cooled (0 °C) solution of *S,S′*-dibenzyl ethylene dicysteine 2HCl (6.0 g) dissolved in 10% K_2_CO_3_ solution (150 mL), and the reaction was stirred for 2 h at 0 °C followed by stirring for 16 h at 25 °C. The solution was extracted with diethyl ether and the crude product precipitated by acidification of the aqueous phase. The product was redissolved and extracted using ethyl acetate to yield an amorphous solid product *N,N′*-dibenzyloxycarbonyl-*S,S′*-dibenzyl ethylene dicysteine (CBz-Bn-EC) (70% yield) upon drying.

CBz-Bn-EC (1.34 g) was activated by reaction with ethyl chloroformate (0.41 g, 2.0 eq) in chloroform (30 mL) with triethylamine (0.38 g, 2.0 eq) at − 15 °C for 15 min. To this reaction mixture, a solution of tetra-acetylglucosamine (1.58 g, 2.2 eq) and triethlyamine (0.42 g, 2.0 eq) in chloroform (30 mL) was added, and the combined reaction mixture was stirred for 1 h at 0 °C and then 12 h at 25 °C. Following an acid–base workup, the residue was purified by column chromatography (MeOH/EtOAc/hexane = 1:35:64 v/v ratio) to afford the fully protected ethylenedicysteine deoxyglucosamine (FP-ECDG) (70% yield).

FP-ECDG (1.0 g) underwent Birch reduction in liquid ammonia (80 mL) with sodium metal (0.5 g). The deep-blue-colored solution was stirred for 20 min at room temperature before quenching with ammonium phenylacetate (1.32 g, 12.0 eq). The formed solution was then dried under argon gas. The ammonium phenylacetate was extracted by stirring twice with isopropanol (50 mL and 25 mL) and separating using centrifugation (5 min, 4000 rpm). Residual isopropanol was removed by stirring and centrifuging with diethyl ether (2 × 50 mL), and the solid product was then dried under argon gas to afford ethylenedicysteine deoxyglucosamine (ECDG) (53% yield). The resultant product was confirmed MS and NMR analysis in accordance with the literature data (16). Calculated m/z 590.665 for C_20_H_38_O_12_N_4_S_2_ [M + H] = 591.1.

### ECDG Kit Preparation

The ECDG kit preparation was done in a one vial procedure according to Zeevaart et al. [17]. All solutions (HCl (0.1 M), Na_2_HPO_4_ phosphate/citrate buffer solution and SnCl_2_ solution (1 mg/mL—ensuring the solution is clear and not milky), were freshly prepared with ultrapure, Milli-Q grade (> 18 MΩ/cm), and degassed water before production of the kits. Once prepared, all water and solutions were filtered through a sterile Millex-GP (polyethersulfone, 0.22 µm, 33 mm) syringe filter (Merck, Massachusetts, USA) into sterilized vials.

Citric acid (0.20 g) was added to a sterile vial containing Na_2_HPO_4_ (0.284 g) dissolved in water (pH 5.5) with the addition of SnCl_2_ solution (100 µL) and freeze-drying (Christ Alpha I-5 freeze-drier, Type 1050 (Medizinische Apparatebau, Harz, Germany) overnight. The ECDG (5 mg) was weighed into a vial under Ar (g) and dissolved in MeOH (0.75 mL). This solution was immediately transferred to the vial containing the Sn/buffer and flash frozen in liquid nitrogen followed by lyophilization overnight. The kits were sealed, capped, and placed in the − 80 °C freezer for storage.

### Synthesis of ^99m^Tc-ECDG

Radiosynthesis of ^99m^Tc-ECDG was completed according to Zeevaart et al. [17] by adding Tc-99 m pertechnetate (^99m^TcO_4_ −) (50–60 mCi) to the prepared lyophilized ECDG kit vial (5 mg ECDG, citric acid/Na_2_HPO_4_, SnCl_2_). The solution was heated at 75 °C for 15 min.

**Fig. 1:**
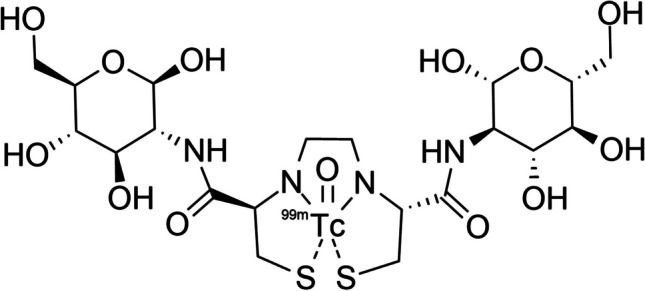
Structure of ^99m^Tc-ethylenedicysteine-deoxyglucose (^99m^Tc-glucosamine)

Quality control was performed on the radiolabeled product by testing pH (pH 5.5) and radiochemical purity (RCP) (> 95%). RCP was determined using HPLC (Varian Prostar 325 UV/Vis (Varian Inc.) fitted with a radiometric GABI Star gamma detector (Raytest GmbH, Straubenhardt, Germany) and thin layer chromatography (TLC) (Raytest GmbH, Straubenhardt, Germany). HPLC analysis was performed using a C-18 reverse phase column (Agilent Luna-C18 column, 5 µm, 4.6 × 250 mm) with isocratic elution (4.5% MeCN in 2 mM ammonium formate (pH 3)) over 35 min. TLC analysis was completed using ITLC-SG (Agilent, USA) and Whatman (Millipore Sigma, USA) paper strips (10 cm) developed with saline and acetone as the mobile phase, respectively. Each strip was cut in half, and the activity was measured at the strip origin and front to determine the percentage of colloids (ITLC-SG) and percentage of labeled product (Whatman). The stability of the ^99m^Tc-ECDG was determined up to 5 h after preparation using HPLC. The structure of ^99m^Tc-ECDG is shown in Fig. 1.

### Radiopharmaceutical Administration

An aseptic dispensing process was followed for the preparation of ^99m^Tc-ECDG patient dose. The sterility of the process was checked using air settle plates and finger dab plates which were cultured. The ^99m^Tc-ECDG dose was prepared for injection by diluting the prepared radiolabeled solution with saline (approx. 3 mL) and filtering through Millex-GP, a sterile filter (polyethersulfone, 0.22 µm, 33 mm, Merck, Massachusetts, USA) into a sealed sterile vial. Additional saline (sterile, 1 mL) was added to the vial to ensure a final volume of around 5 mL. A patient dose of 20–25 mCi was withdrawn for intravenous administration. Fasting was not a requirement prior to the administration of the radiopharmaceutical. No adverse events were recorded after radiopharmaceutical administration.

### Imaging Protocol

All 22 participants were scanned using a dual-head gamma camera (Siemens Symbia T16 True point SPECT-CT; Siemens medical solutions, USA). The SPECT/CT camera was equipped with a low-energy, high-resolution collimator (LEHR). Dynamic images of the clinically most symptomatic joints were acquired at the time of administering the radiopharmaceutical, with a frame rate of 1 fps for 60 s. This was followed by blood pool imaging of the hands, wrists, and knees. A delayed whole body image was acquired 2 h after radiotracer injection, followed by dedicated 5 min static images of the hands, wrists, and knees. SPECT images of the most clinically symptomatic joint (either the hands/wrist or knees) were also performed at 25 s/stop, with 3° steps, in a 128 × 128 matrix. This was followed by a low dose, non-contrast CT, with the patient in the same bed position.

### Image Processing and Data Analysis

Images were processed using the Syngo workstation on the gamma camera. SPECT images were reconstructed using an iterative algorithm and SPECT/CT fusion images were obtained using the multimodality Syngo imaging software on the workstation.

The data of each patient were collected using an Excel 2019 spreadsheet (Microsoft, USA). Statistical analysis was performed using R, version 4.3.0 (R Foundation for Statistical Computing, Vienna, Austria).

### Image Interpretation

Images were interpreted by a single nuclear medicine physician with 9 years experience. The dynamic flow images were assessed qualitatively for an increase, a decrease, or normal blood flow to the region imaged. The blood pool images were assessed for increased, decreased, or normal blood pool activity. Delayed images were interpreted for disease activity in the joints, using a slight modification of the scoring system used by Angelides et al. [14]:

- Grade 0—Normal physiological joint uptake, defined as no/minimally increased radiotracer activity in joints (activity same as that of the neighboring muscle tissue).
- Grade 1—Mild radiotracer uptake slightly more than that of the neighboring muscle tissue.
- Grade 2—Moderate radiotracer uptake greater than that of grade 1.
- Grade 3—Severe radiotracer uptake markedly greater than that of grade 1.

The whole body images were qualitatively assessed for the biodistribution of the radiotracer.

## Results

The ECDG ligand was prepared in 5 steps (overall yield of 8%) and then successfully formulated into buffered kits which were stably stored in a − 80 °C freezer. Radiolabeling of the ECDG kit with ^99m^TcO_4_ − saline yielded ^99m^Tc-ECDG (10–12 mCi/mg) in 97% radiochemical purity as determined by radio-HPLC analysis. ^99m^Tc-ECDG presented as 4 diastereomeric peaks with retention times of 6.5, 8.5, 10.4 and 11.4 min. TLC analysis indicated almost no colloid formation (< 0.2%) and > 98% labeled product. The radiolabeled product formulation was stable over 5 h at room temperature with no visible degradation and only a 1.5% loss of bound radioactivity.

Twenty-two participants with diagnosed RA according to the ACR/EULAR classification criteria were recruited into the study. The median (IQR) age was 59 (49–68) years, and the majority (95.5%) were females. Twelve participants had the joints of their hands and wrists clinically examined, as this was their most symptomatic joints, while the remaining ten had their knees clinically examined. So, a total of three hundred and thirty-six joints in both hands, which included the metacarpophalangeal (MCP) joints, proximal interphalangeal (PIP) joints, and distal interphalangeal (DIP) joints, were evaluated for disease activity. Twenty-four carpal joints and twenty knee joints were also assessed for disease activity. Therefore, a total of 380 joints were evaluated for disease activity.

**Fig. 2:**
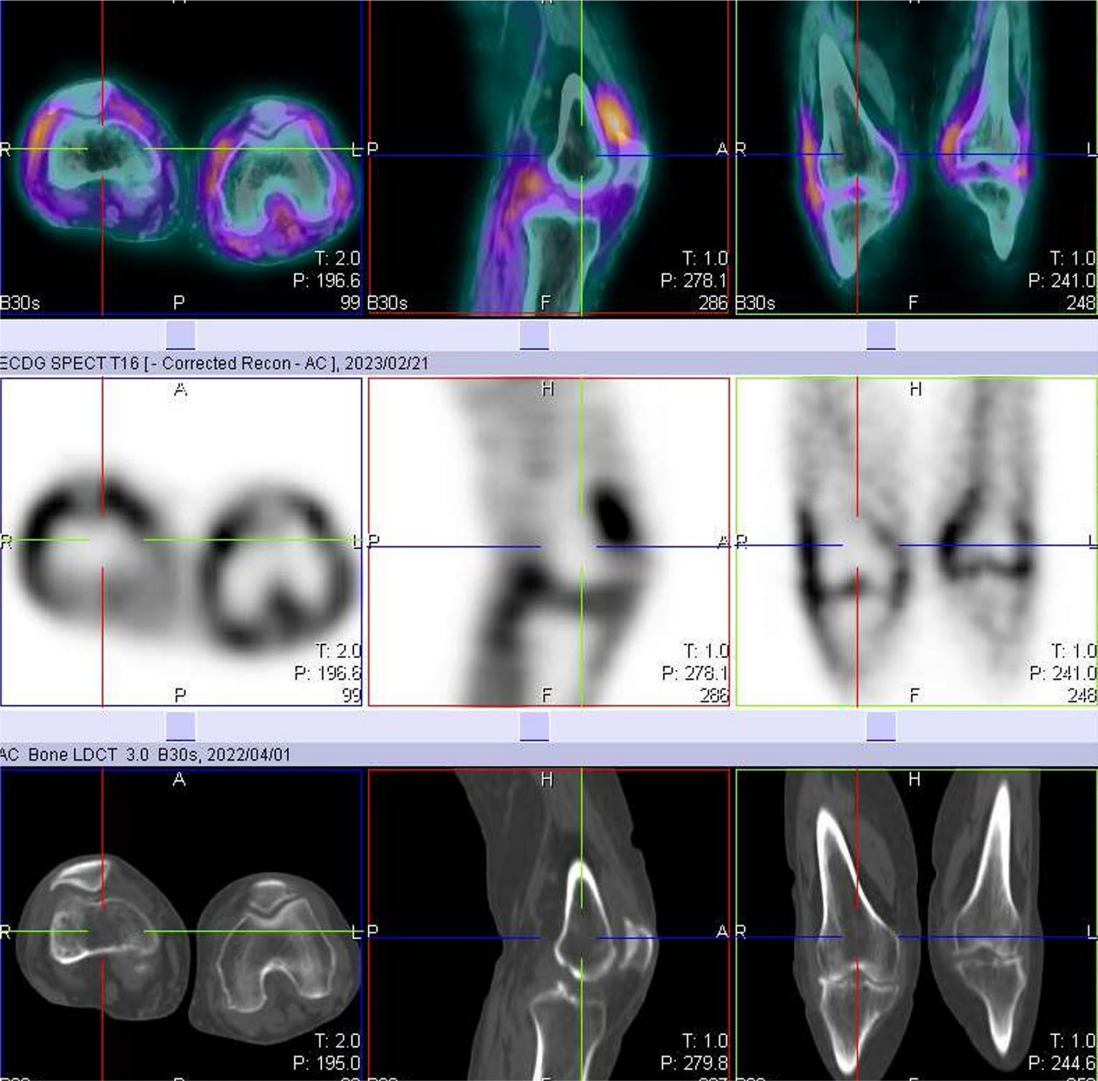
SPECT/CT, SPECT, and CT only images of an 82-year-old female with rheumatoid arthritis of both knees. Note the localization of radiotracer to the synovial space, without bone involvement

**Fig. 3:**
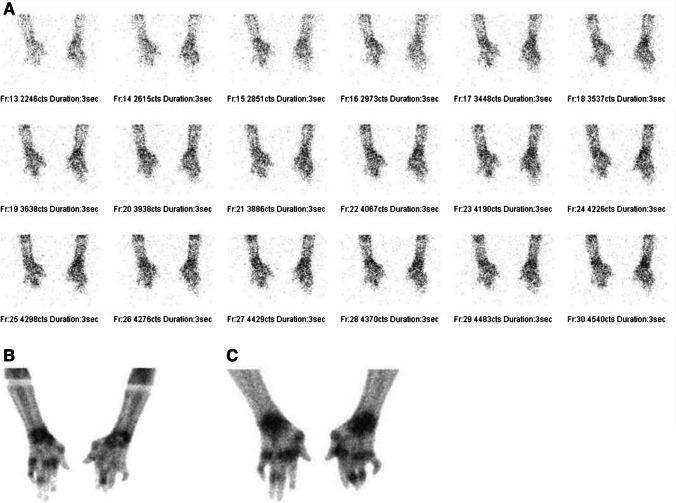
Three phase ^99m^Tc glucosamine scan of a 58-year-old female with rheumatoid arthritis that developed a flair after discontinuing her medications. The flow images (**A**) show increased blood flow to the region of the wrists and small bones of the hands. The blood pool and delayed static images (**B** and **C**, respectively) show increased blood pool and delayed activity to the wrists and small joints of the hands, respectively

**Fig. 4:**
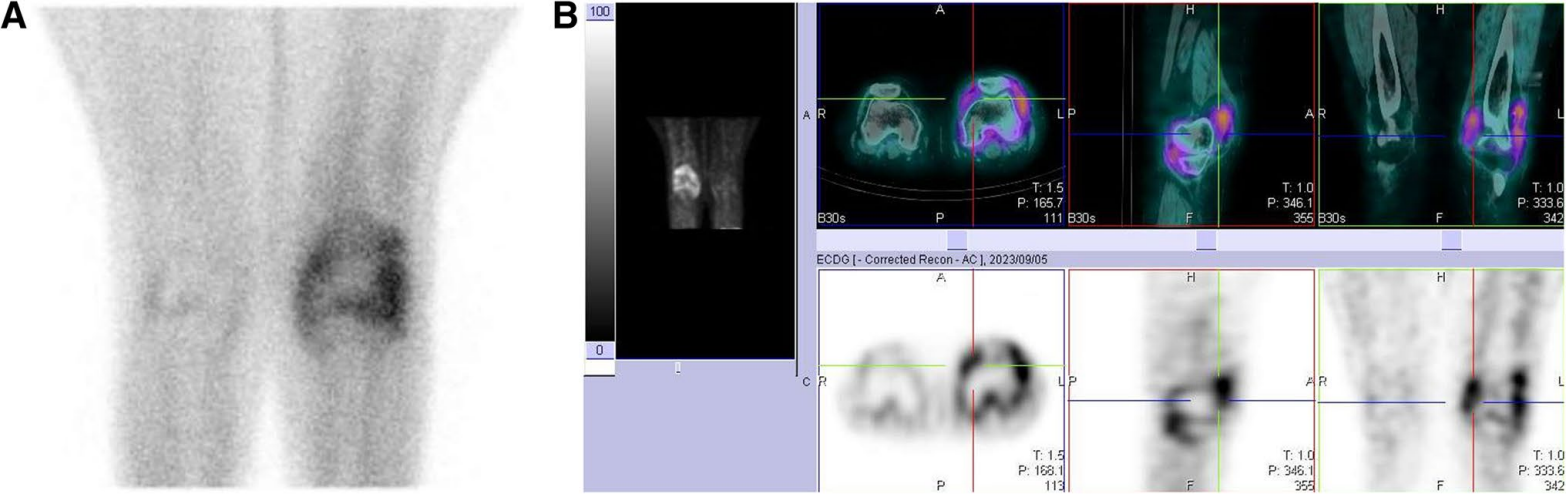
Delayed static (**A**) and SPECT/CT images of a 51 year old with unilateral knee disease. Note the normal physiologic uptake of radiotracer in the right knee

Overall, the study was well tolerated, with none of the patients presenting with any adverse events. All 22 participants had abnormal increased uptake of the radiopharmaceutical in their affected joints, with SPECT/CT imaging localizing uptake specifically to the synovial space as seen in Fig. 2. Of the 22 flow studies, 10 (45%) participants had normal flow to their most symptomatic joints, with 12 (55%) having increased flow to their most symptomatic joints. Increased flow studies were associated with either grade 3 or 2 disease on the delayed static images as shown in Fig. 3. Good quality images were obtained 2 h post ^99m^Tc-ECDG administration, with an optimal target to background ratio. The majority of the participants had the greatest uptake of radiotracer in their most clinically active joints (*n* = 15, 68%). The mean DAS-28 score for all the participants was 41, with a range of 17–67. Majority of the participants had high DAS-28 scores indicating severe disease as seen in Table 1 showing the characteristics of the study population. All participants with clinically severe disease had an overall grade 3 or 2 uptake of ^99m^Tc-ECDG on their scans, with 55% being grade 3 uptake. Twelve participants had evidence of disease in other joints outside the hands, wrists, and knees. These joints included the shoulders (*n* = 9), elbows (*n* = 4), and ankles (*n* = 2). Two participants presented with unilateral knee disease, as seen in Fig. 4. No incidental finding of extra articular disease was noted in any of the 22 participants.

**Table 1:** Characteristics of the study participants (*n* = 22)

Variable	
Age in years, median (IQR)	59 (49–68)
Sex, *n* (%)	
Female	21 (95.5)
Male	1 (4.5)
DAS-28 severity, *n* (%)	
Mild	0 (0)
Moderate	2 (9.1)
Severe	20 (90.9)
^99m^Tc-ECDG overall uptake grade, *n* (%)	
Grade 1	2 (9.1)
Grade 2	8 (36.4)
Grade 3	12 (54.5)
Serological markers, median (IQR)	
Anti-CCP (normal/reference value < 3 U/mL)	63 (2.6–201)
Rheumatoid factor (RF) (normal/reference value < 20 IU/mL)	38 (11–81)
Positive (elevated) RF and anti-CCP antibody titers, *n* (%)	
Yes	14 (63.6)
No	8 (36.4)

*IQR* interquartile range, *anti-CCP* anticyclic citrullinated peptide, *U/mL* units per milliliter, *IU/mL* international units per milliliter

Comparing the detection of disease activity by clinical evaluation and ^99m^Tc-ECDG SPECT/CT imaging, interestingly, all 12 participants who have the joints of the hands evaluated with both modalities had no detection of disease activity by both modalities in the 96 DIP joints. However, there were some discrepancies in the other 240 joints evaluated, as seen in Table 2. The level of agreement, expressed as percentages in the detection of synovitis in the hands, wrists and knees, was 56.2, 75, and 85% respectively as seen in Tables 2, 3, and 4.

**Table 2:** Percentage of active disease detected in the joints of the hands by both modalities and their agreement

Modality	Number of joints assessed	Positive for disease, *n* (%)
Clinical examination	240	152 (63.3%)
^99m^Tc-glucosamine SPECT/CT	240	121 (50.4%)

Percentage agreement (tolerance = 0) = 56.2

**Table 3:** Percentage of active disease detected in the joints of the wrists by both modalities and their agreement

Modality	Number of joints assessed	Positive for disease, *n* (%)
Clinical examination	24	20 (83.3%)
^99m^Tc-glucosamine SPECT/CT	24	22 (91.7%)

Percentage agreement (tolerance = 0) = 75

**Table 4:** Percentage of active disease detected in the joints of the knees by both modalities and their agreement

Modality	Number of joints assessed	Positive for disease, *n* (%)
Clinical examination	20	18 (90.0%)
^99m^Tc-glucosamine SPECT/CT	20	19 (95.0%)

Percentage agreement (tolerance=0) =85

**Fig. 5:**
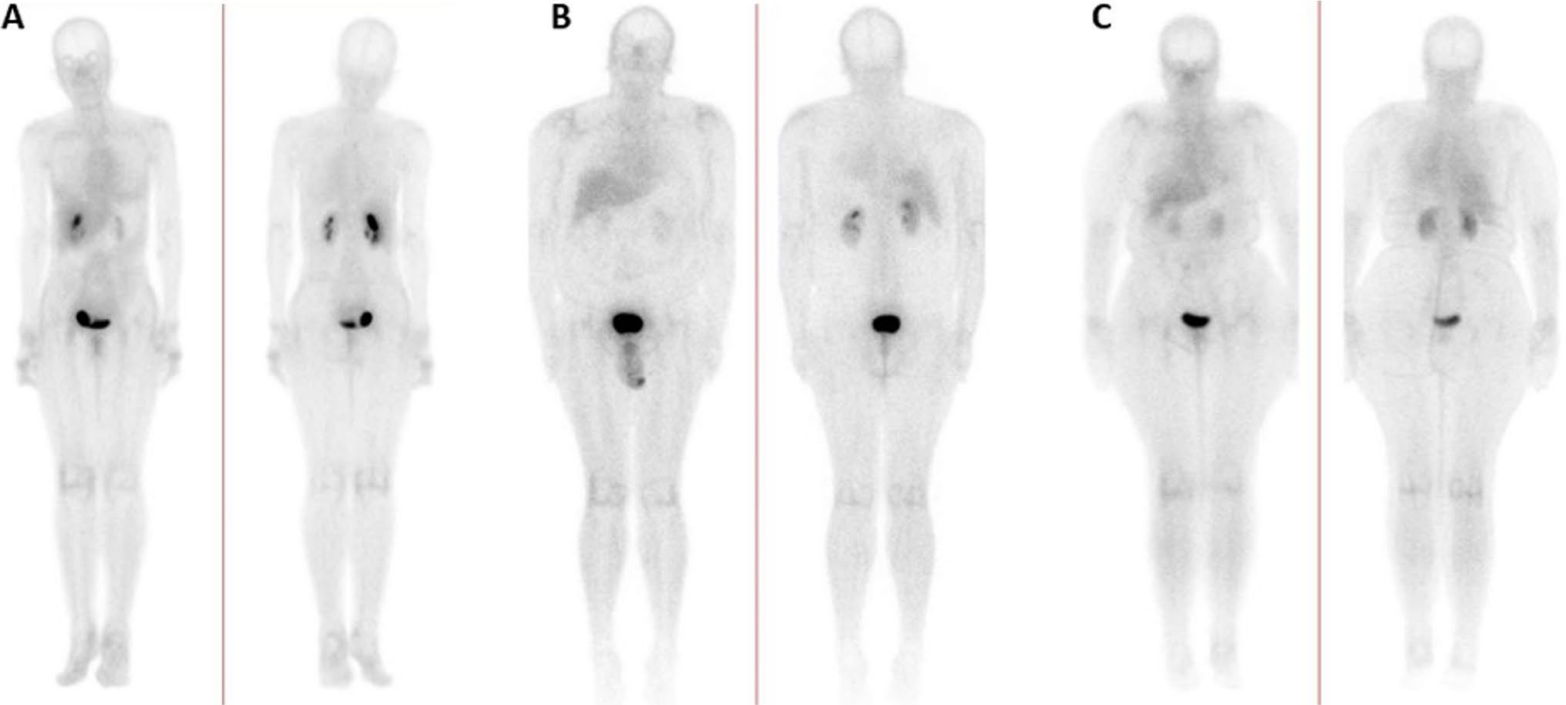
Whole body images of a patient with severe disease (**A**), mild disease (**B**), and a non-fasted patient (**C**). Note the similarities in the biodistribution

Irrespective of disease activity or fasting state, the general biodistribution of ^99m^Tc-ECDG was similar on the delayed 2 h whole body images in all 22 participants. Very mild uptake was noted in the unaffected joints, spine,, and skeletal muscle, with occasional mild uptake visible in the lacrimal gland and nasal mucosa. Mild to moderate uptake was noted in the blood pool and liver. The radiopharmaceutical exhibited renal excretion, with moderate activity noted in the kidneys and very intense uptake noted in the urinary bladder. None of the patients had uptake of ^99m^Tc-ECDG noted in the brain, lungs, and myocardium. Figure 5 shows the distribution of ^99m^Tc-ECDG in a participant with high disease activity, mild disease activity, and in a known non-fasted participant.

## Discussion

The findings indicated that most of our participants had increased uptake of ^99m^Tc-ECDG in their clinically affected joints, with varying degrees of uptake. This is not different from the few studies that have used ^99m^Tc-ECDG to evaluate disease activity in patients with RA [6, 14]. However, the level of agreement with the two modalities (clinical evaluation and ^99m^Tc-ECDG), expressed as percentages in the detection of synovitis in the knees, wrists, and hands, was 85, 75, and 56.2%, respectively. This finding was largely due to the fact that ^99m^Tc-ECDG imaging detected disease activity in some sites that were negative for disease activity by clinical evaluation, especially in the joints of the hands. This is likely in keeping with its ability to detect subclinical disease as noted in an earlier study and the fact that clinical evaluation is very subjective [14]. There was however a good correlation between the degree of severity clinically using the DAS-28 scores and the grade of ^99m^Tc-ECDG uptake.

Our findings also show that all the participants showed similar biodistribution of the radiopharmaceutical. This is also similar to the first study assessing biodistribution, in which the biodistribution of ^99m^Tc-ECDG was evaluated in patients with non-small lung cell cancer [18]. Just like the study published by Angelides et al. [14], ^99m^Tc-ECDG was well tolerated by all our participants, with no allergic reactions or side effects. In our study, majority of the participants had the greatest uptake of radiotracer in their most clinically active joint. This is also almost similar to the findings of the previously published study by Angelides et al. [14], where the highest radiotracer uptake was noted in the most clinically affected joint. However, even though majority of our participants had these findings, 32% of them did not. This might be attributable to one of the draw backs of clinical estimation of disease activity, whereby its subjective nature might reduce its diagnostic accuracy [19]. Further observations from our study included that apart from symptomatic joints, disease activity was also noted in non-symptomatic joints, including joints outside the hands, wrists, and knees. A finding that is also similar to that published by Angelides et al. [14]. We agree that this finding might account for subclinical disease, an entity that is known to be associated with disease progression in otherwise healthy looking patients. This is likely to be an advantage of ^99m^Tc-ECDG imaging, as it can detect patients with subclinical disease. Our findings are also similar to the published study by Manolois et al. [6], who imaged 25 patients with RA, and concluded that the radiopharmaceutical was well tolerated, and its degree of uptake demonstrated significant correlation with clinically assessed disease activity. They also concluded that it also accumulates in sites of subclinical disease, which is similar to our findings. Both of these previously published studies reported that ^99m^Tc-ECDG is similar to [^18^F]FDG, and being analogues of glucose, they are metabolized by inflamed tissue. This was based on the information from Yang et al. [20, 21] that ^99m^Tc-ECDG acts as a glucose analogue and can also be used in the imaging of malignancies. Various authors have also tried to assess the imaging properties of ^99m^Tc-ECDG in malignancies. Ginat et al. [22], in their study of 9 patients with head and neck squamous cell carcinoma, confirmed increased uptake of the radiotracer in all 9 cases. In a pre-clinical study conducted by Pham et al., they concluded that this radiopharmaceutical was easily taken up both in vivo and in vitro in murine cells by diffuse large B cell lymphoma (DLBCL) cells [23]. However, we had imaged one patient with DLBCL in our facility, known with multiple FDG avid lymph nodes and nodal masses above and below the abdomen, but these lesions had no uptake of ^99m^Tc-ECDG. Other studies have also investigated the potential utility of this radiopharmaceutical for tumor imaging. Zhang et al. in their study investigated mesothelioma-bearing rats and confirmed increased uptake of ^99m^Tc-ECDG in the tumor [24]. A published study of 17 patients with non-small cell lung cancer compared ^99m^Tc-ECDG and [^18^F]FDG, in the imaging of these patients [25]. They concluded that there was a 100% concordance in the imaging of the primary tumor and 70% concordance in the imaging of metastatic disease with the two radiotracers. All these studies seem to support the idea that ^99m^Tc-ECDG, being an analogue of glucose, is metabolized and taken up by tumor cells, with some alluding to the fact that it shares similar properties with [^18^F]FDG. However, most of these studies are hampered with the fact that they are either pre-clinical studies or studies involving a very small sample size. Therefore, it cannot still be concluded that this radiopharmaceutical behaves like [^18^F]FDG.

Findings from this RA study may suggest that ^99m^Tc-ECDG and [^18^F]FDG do not show similar properties. In our study, we noticed that, irrespective of the fasting state of the patient, the biodistribution of the radiopharmaceutical remained the same, which is not the case with [^18^F]FDG. The biodistribution of [^18^F]FDG is associated with intense uptake in the brain, as well as uptake in the myocardium and bone. None of our participants exhibited ^99m^Tc-ECDG uptake in these areas, although we noticed very mild uptake in the spine of most of the participants. These findings are relatively similar to those published by Schechter et al. [18]; however, they postulated that the hydrophilic nature of the radiopharmaceutical might prevent it from crossing the blood brain barrier. This, however, does not explain the absence of uptake in the bone and myocardium.

Although not yet proven, the information obtained from our study has led us to the hypothesis that the uptake of ^99m^Tc-ECDG in the affected joints of our participant population might not be due to its similar properties to [^18^F]FDG, but to the fact that glucosamine is a normal constituent of synovial fluid and that there may be upregulation of glucosamine receptors in the synovium of patients with RA. Further investigation might be required to conclude on the possibility of this hypothesis. But what is clear is that ^99m^Tc-ECDG has different imaging properties as compared to [^18^F]FDG but could be considered as a good alternative for imaging RA in centers that have only SPECT/CT scanners and no PET/CT scan scanners. Overall, the findings of this study are similar to the few previously published studies in the imaging of patients with RA; however, a significant limitation of this study is the small sample size. Larger patient numbers would be required to increase the weight of any claims. The varying degree of uptake in our participant population could signify that this radiotracer might be valuable in assessing treatment response and offering prognostic information. We recommend that further studies should be conducted in this regard.

## Conclusion

This study has shown that ^99m^Tc-ECDG (^99m^Tc-glucosamine) is a safe radiotracer that can efficiently assess active disease activity in the joints of patients with RA. It accumulates in sites of both clinical and subclinical disease and might be a very useful tool for the rheumatologist in the management of patients with RA. The study also showed a good correlation between disease severity clinically and degree of ^99m^Tc-ECDG uptake. ^99m^Tc-ECDG offers centers with only SPECT or SPECT/CT scanners an opportunity to investigate patients with RA.

## Data Availability

Contact the corresponding author for data requests.

## Declarations

### Author Information

Dr. Evbuomwan Osayande is a nuclear medicine specialist and a senior lecturer.

Barend Jansen Van Rensburg is a rheumatologist and a senior lecturer.

Dr. Engelbrecht Gerrit is a nuclear medicine consultant and head of department of nuclear medicine.

Dr. Cathryn Driver is a senior scientist in radiochemistry and molecular imaging.

Prof. Mathys Labuschagne is a professor in health professions education and an ophthalmologist.

Joseph Sempa is a biostatistician and senior lecturer.

Dr. Je'nine Horn-Lodewyk is a PhD holder and medical imaging technologist.
